# Early efficacy of stereotactic body radiation therapy combined with adoptive immunotherapy for advanced malignancies

**DOI:** 10.3892/mco.2013.157

**Published:** 2013-07-23

**Authors:** YI-SHAN WANG, GUIQING YANG, YUAN-YUAN WANG, JIA-LIN YANG, KE YANG

**Affiliations:** Center for Tumor Treatment, People’s Liberation Army 107th Hospital, Yantai, Shandong 264003, P.R. China

**Keywords:** CyberKnife, cytokine-induced killer, optimal treatment, advanced malignant tumor, combined modality

## Abstract

Stereotactic body radiation therapy (SBRT) concentrates radiation to a predefined target, affecting all the cells within it. Adoptive immunotherapy is not restricted by the major histocompatibility complex (MHC) in recognizing and eliminating target cells. We investigated the effects of the combined modality of SBRT and adoptive immunotherapy on patients with advanced malignant tumors. The database of 316 patients with 845 tumors who underwent SBRT between April, 2010 and February, 2012 was retrospectively reviewed. Of the 316 patients, 145 received biological immunotherapy and were assigned into the observation group, whereas the remaining patients constituted the control group. Patients in the two groups were recorded on efficacy assessment, Karnofsky performance status (KPS), cell phenotype expression level *in vitro* and the percentages of lymphocyte subsets and ratio of CD4^+^/CD8^+^ lymphocytes in the peripheral blood. Following treatment, the total effectiveness [complete response (CR) + partial response (PR)], the KPS score, the percentages of lymphocyte subsets and the CD4^+^/CD8^+^ lymphocyte ratio in the observation group were higher compared to those in the control group, with a statistically significant difference (P<0.05). The expression of CD3^+^ and CD3^+^CD56^+^ cytokine-induced killer (CIK) cells were increased from 56.76±4.54% and 11.32±2.96% to 94.67±4.46% and 32.65±1.12%, respectively, when cultured *in vitro* (P<0.01). The percentages of lymphocyte subsets and the CD4^+^/CD8^+^ lymphocyte ratio were significantly increased compared to prior to treatment in the observation group (P<0.05). SBRT combined with adoptive immunotherapy may be a novel therapeutic option for patients with advanced malignant tumors.

## Introduction

Advances in the diagnosis of various solid tumors is reflected by an increasing incidence of malignant tumors. Malignancy is an important cause of human mortality and the most challenging to treat, usually resulting in local or distant metastatic dissemination. The recurrence of cancer is considered an unfavorable situation. The persistence of cancer cells in or around the organ or the primary lesion after surgery and an ineffective anticancer immune response are associated with the majority of patients (1). Treatment of malignant tumors is complicated, aiming to provide local control and preventing the regeneration and diffusion of tumor cells. Therefore, the key to the treatment of malignant cancer is to optimize the balance between local control and systemic immune responses (2). Surgical resection, which provides immediate relief of tumor compression, is the preferred therapy for the majority of solid tumors. However, there are cases that are ineligible for surgery due to a risk of excessive surgical complications, determined by age, performance status, size, volume, location and number of metastases at presentation and status of systemic disease.

Radiotherapy may be important in the treatment of these cases. Unlike traditional radiation therapy, stereotactic body radiation therapy (SBRT) with CyberKnife^®^ converges multiple radiation beams to deliver a single, large dose of radiation to a discrete tumor target with high precision (3). CyberKnife possesses the advantage of precise biological effective dose delivery for target localization, while minimizing irradiation of the surrounding normal tissue (4), playing a critical role, particularly in the treatment of complicated malignant tumors. Cytokine-induced killer (CIK) cells are recently discovered immune effector cells exhibiting potent, non-major histocompatibility complex (MHC)-restricted cytolytic activities against susceptible tumor cells of autologous and allogeneic origins (1,5). CIKs possess versatile antitumor advantages, including easier generation from T cells, more potent cytotoxicity against tumors and more enhanced *in vivo* proliferation potency (6–8). Furthermore, CIK cells were reported to have minimal cytotoxic activity against normal marrow or hematopoietic cells (9). We conducted a randomized controlled trial to assess whether adoptive immunotherapy with CIK cells may be beneficial when used as adjuvant therapy with SBRT in the treatment of malignancies.

## Materials and methods

### Patients

This clinical trial of SBRT CyberKnife and CIK cell-based immunotherapy was approved by the Institutional Ethics Committee and written informed consent was obtained from each patient. The medical records of 316 consecutive patients with a total of 845 malignant tumors that were treated at the Center for Tumor Treatment of People’s Liberation Army 107th Hospital between April, 2010 and February, 2012 were reviewed. The inclusion criteria were: i) pathological or radiographic confirmation of stage III–IV malignant tumors; ii) Karnofsky Performance Status (KPS) ≥50; iii) life expectancy ≥3 months; and iv) informed consent for detection and treatment from all the enrolled patients.

### Research methods

Quantifiable information on the patients was obtained. An enhanced, 1.25-mm slice computed tomography scan was performed prior to CyberKnife (Accuray, Inc., Sunnyvale, CA, USA) treatment. The patients had 2–3 gold fiducial markers implanted in the lung, liver or pancreas under CT guidance. The gross tumor volume (GTV) was determined by CT or MRI. A margin of 0–7 mm around the GTV was added in patients with fiducials to define the planning target volume (PTV). The treatment planning and therapeutic dose were determined according to the number, size and distribution of tumors and the risk coefficient of the patients. A total of 3 or 5 fractions were delivered within 1 week. Most of the PTV was encompassed by the 70–90% isodose line of the prescribed dose. A total median dose of 43 Gy (range, 1,800–6,500 Gy) was administered to the patients, with a dose of 4–18 Gy per fraction.

Peripheral blood (50 ml) was obtained from each eligible patient prior to the CyberKnife (Accuray) treatment. The monocytes separated and extracted from the peripheral blood by centrifugation were then cultured in complete culture media (RPMI DMEM^®^; Beyotime Institute of Biotechnology Co., Ltd., Shanghai, China) + Gentamicin^®^ (Beyotime Institute of Biotechnology Co., Ltd.) + 5% AB Blood Serum^®^ (Beyotime Institute of Biotechnology Co., Ltd.) with 1,000 U/ml of interferon-γ (IFN-γ)^®^ (Beyotime Institute of Biotechnology Co., Ltd.) for 12–14 days. All the cultured CIK cell samples were required to meet the following criteria: i) number of cells >1×10^10^ (10 billion) and percentage of actived cells ≥95%; ii) CD3^+^ cells 79.39–99.28%, CD3^+^CD8^+^ cells 37.12–85.83% and CD3^+^CD56^+^ cells 25.46–44.16%; and iii) the dye-exclusion test was used to exclude possible contamination of the final cell products by bacteria, fungi and endotoxins. FITC-labeled mouse-antihuman CD3 and PE-conjugated mouse anti-human CD8/CD56 monoclonal antibody^®^ (100 ng/ml; Becton Dickinson Medical Devices Co., Ltd., Shanghai, China), interleukin-2 (IL-2^®^, 1000 U/ml; Beijing Double-Crane Pharmaceutical Equipment Co., Ltd., Beijing, China) and IL-1α^®^ (1000 U/ml; Beijing Double-Crane Pharmaceutical Equipment Co., Ltd.) were added to the culture media on days 1 and 14. Following incubation for 20 min at room temperature, the CIK cells were washed twice with PBS^®^ (Beyotime Institute of Biotechnology Co., Ltd.) and analyzed by Flow Cytometry^®^ (Becton Dickinson Medical Devices Co., Ltd.) according to the cellular phenotype prior to CIK cell administration. The mature CIK cells were harvested with sacked normal saline with 3 ml of IL-2 (Beijing Double-Crane Pharmaceutical Equipment Co., Ltd.) and 200 ml of 10% human serum albumin^®^ (Beyotime Institute of Biotechnology Co., Ltd.). No more than 10 billion CIK cells were administered to the patients at one time, with each course comprising two administrations. Clinical treatment schemes are provided in Table I.

### Evaluation and statistical analysis

Evaluations were performed 1 month after the CIK therapy and every 3 months thereafter. The median follow-up period was 10 months (range, 6–18 months). Regular subsequent studies included reexamination of MRI or CT scans, assessment of KPS and evaluation of cell phenotype expression levels *in vitro* and percentages of lymphocyte subsets prior to and following CIK cell therapy. The objective curative effects between the two samples were analyzed using the Chi-square test. The comparison of cell phenotype prior to and following CIK cell culture were performed with the paired t-test. Lymphocyte percentages prior to and following CIK cell therapy were assessed by the paired samples t-test, lymphocyte percentages and KPS scores between observation and control groups were compared with the independent samples t-test. P<0.05 was considered to indicate a statistically significant difference. SPSS 13.0 software was used for statistical analysis.

## Results

A total of 316 patients with malignant tumors were treated with CyberKnife and 145 patients received additional CIK cell therapy: 97 patients received 5 courses of CIK treatment, 36 received 8 courses and 12 received 9 courses.

### Response assessments

According to the general standard for solid tumors (WHO), complete response (CR) was defined as disappearance of all target lesions measured by MRI or CT, partial response (PR) was defined as a decrease in the sum of the maximum diameter (MDs) of the target lesions by ≥30%, progressive disease (PD) was defined as an increase of 20% in the sum of the MDs of the target lesions and stable disease (SD) was defined as insufficient shrinkage or increase of the tumorous lesions to qualify for PR or PD, respectively. The total effectiveness rate was calculated as: (CR + PR)/total number of cases × 100%. The total effectiveness rate of the observation group was 66.8% and that of the control group 60.2%. The two groups exhibited a statistically significant difference in clinical curative effect (P<0.05). The KPS scores in the two groups were higher compared to prior to treatment and the KPS score of the observation group was higher compared to that of the control group, with a statistically significant difference (P<0.05). Flow cytometric analysis demonstrated an increase in the expression of CD3^+^ and CD3^+^CD56^+^ CIK cells from 56.76±4.54% and 11.32±2.96% to 94.67±4.46% and 32.65±1.12%, respectively, when cultured *in vitro* (P<0.01). Preliminary clinical data are presented in Table II.

Peripheral blood samples were collected from patients who underwent CIK immunotherapy and the changes in the lymphocyte subsets 1 month after the patients received CIK cell infusions were measured. The percentages of CD3^+^, CD3^+^CD8^+^, CD3^+^CD4^+^ and CD16^+^CD56^+^ cells and the CD4^+^/CD8^+^ ratio were higher compared to prior to treatment in the observation group. The overall populations of CD3^+^, CD3^+^CD8^+^, CD3^+^CD4^+^ and CD16^+^CD56^+^ cells and the CD4^+^/CD8^+^ ratio were significantly lower in the control group compared to that in the observation group. The results are presented in Table III.

## Discussion

Palliative treatment is often applied to patients with high-risk or malignant tumors due to their ineligibility for surgery and conventional radiation or chemotherapy (10,11). In the past, patients with inoperable advanced malignant tumor were chiefly referred for traditional radiation therapy which exerted a profound toxic effect on the surrounding tissues and was likely to raise the risk of recurrence or further deterioration. With the advent of modern CyberKnife technology, there has been a renewed interest in radiation. CyberKnife is a versatile method with the advantages of high fraction dose, high precision and timely tracking. Rapid dose delivery reduces the risk of radiation damage to surrounding tissues and the high doses may be able to overcome the radioresistance of refractory tumor cells. CyberKnife combines the mechanical strength of stereotaxy with the radiobiological advantages of fractionation and overcomes severe complications resulting from single high-dose stereotactic radiosurgery treatment (12).

Although technical advances in the diagnosis and the treatment of malignant tumors have achieved significant improvements in survival, the incidence and mortality rates are currently on the increase worldwide. Tumor cells may produce a certain soluble protein, thus escaping from the immune surveillance of the tumor-bearing host (13). The role of the immune system is to protect the body. This protective function is performed by leukocytes and a number of accessory cells distributed throughout the body. Immunotherapy based on dendritic, lymphokine-activated killer (LAK), natural killer (NK), cytotoxic T and CIK cells may eliminate cancer cells and enhance antitumor immunity (13). Since cytokines are key elements of the host immune response, adoptive CIK cells play an important role in immunotherapeutic cancer treatment.

Tumor-specific cytotoxic T cells may be isolated from the peripheral blood. NK cells activated by IL-2 are cytotoxic cells responsible for cellular cytotoxicity. As the major effector population in LAK cells, NK cells actively increase autoproteolysis of cancer cells. The higher lytic activity of CIK compared to LAK cells is mainly due to the higher proliferation of CD3^+^CD56^+^ cells and the cytotoxic activity of T-cell receptors (TCRs) (8). CD3^+^CD56^+^ NK cells are rare in uncultured human peripheral blood. They become the predominant part of CIK cells and are generated *ex vivo* by incubation of peripheral blood lymphocytes with an agonistic anti-CD3 monoclonal antibody (14). CD3^+^CD56^+^ cells may increase up to 1,000-fold and exhibit the highest cytotoxicity against various tumor cell targets (1). During the generation of CIK cells, the total number of CD56^+^ cells increases, mainly due to the expansion of CD56^+^ cells coexpressing CD3. Our culture system increased the total number of potential effectors. In our study, the percentage of CD3^+^ cells cultured *in vitro* reached 94.67±4.46%, suggesting that the majority of adoptive cells are T cells. The CD3^+^CD56^+^ NK T-cell percentage was increased to 32.65±1.12%, in accordance with the percentage previously mentioned in the literature, which was ≥30% (15). This finding indicates that all these cytokines are able to increase the human body anticancer ability.

The higher number of CD3^+^ T lymphocytes is probably associated with the antitumor effect (16). A previous study by Maluf *et al*(17) suggested that CD4^+^ T lymphocytes may regulate the cellular as well as the humoral immune response, through induction or inhibition. It was demonstrated that endogenously reconstituted and exogenously restored antiviral CD8^+^ T lymphocytes play a protective role in cytomegalovirus aplastic anemia in mice caused by hematoablative treatment with 6 Gy of irradiation (18). Increasing the CD4^+^/CD8^+^ T-cell ratio may improve the antitumor ability and is associated with a reduced risk of tumor recurrence (19). The lymphocyte subgroups CD3^+^, CD4^+^ and CD8^+^ and the CD4^+^/CD8^+^ T-cell ratio may be valuable indices for the prediction of the prognosis of patients with malignant tumors. The persistence and expansion of the infused cells *in vivo* is the fundamental determinant of the therapeutic potency of adoptive T-cell transfer. In our study, the changed trend of each immune index of lymphocyte subsets in the peripheral blood was compared in the observation group. The percentages of CD3^+^, CD3^+^CD8^+^, CD3^+^CD4^+^ and CD16^+^CD56^+^ cells and the CD4^+^/CD8^+^ ratio were significantly increased (P<0.05). The differences in each immune index of lymphocyte subsets were compared between the observation and control groups. The overall populations of CD3^+^, CD3^+^CD8^+^, CD3^+^CD4^+^ and CD16^+^CD56^+^ cells and the CD4^+^/CD8^+^ ratio were significantly lower in the control group compared to those in the observation group (P<0.05). As regards the biological characteristics of immunotherapy, our study revealed a significant correlation with the lymphocyte subsets. The increases in lymphocyte subsets in the peripheral blood are probably responsible for the antitumor effect. However, the correlation with the response and whether it is associated with the prevention of metastasis and recurrence, the improvement of prognosis and the prolongation of survival, must be elucidated by further studies.

Currently, there are several ongoing trials on the treatment of malignant tumors with CyberKnife or CIK cells. However, assessments of the combination of CyberKnife and CIK immunotherapy in the treatment of patients with malignant tumors are limited. Our study evaluated the effects of CyberKnife or the combination of CyberKnife and CIK treatment on patients with malignant tumors. The total effectiveness rate (CR + PR) was 66.8% in the observation group compared to 60.2% in the control group (P<0.05). Following treatment, the KPS scores in the two groups were higher compared to prior to treatment and the KPS score of the observation group was higher compared to that of the control group, with a statistically significant difference (P<0.05). This study has demonstrated that the combination of CyberKnife and CIK immunotherapy may eliminate malignant cells synergistically, confirming the efficacy of this treatment in cancer patients.

## Figures and Tables

**Table I:** Clinical treatment schemes.

Diseases	Patients (no.)	Tumor (no.)	Fractions (no.)	Total dose (Gy)	Patients with CIK cell treatment (no.)
Peripheral lung cancer	53	144	3–5	40–60	23
Central lung cancer	44	137	3–5	30–40	22
Liver cancer	65	152	3–5	25–45	40
Pancreatic cancer	44	59	3–5	25–35	23
Renal cell carcinoma	17	26	3–5	30–40	8
Prostate cancer	6	6	3–5	30–40	2
Malignant glioma	9	56	3–5	30–45	3
Brain metastatic tumor	31	145	3–5	30–40	9
Pituitary tumor	11	15	3–5	25–35	4
Vertebral body tumor	36	105	3–5	30–35	11

CIK, cytokine-induced killer.

**Table II:** Preliminary clinical data (means ± SD).

Variable	Observation group (%)	Control group (%)
Efficacy assessment (WHO)	66.8	60.2
KPS		
Prior to treatment	65.73±9.05	64.00±9.00..
Following treatment	83.36±11.64	71.64±10.60
Phenotype of CIK cells prior to transfusion		
CD3^+^		
Prior to culture	56.76±4.54	-
Following culture	94.67±4.46	-
CD3^+^CD56^+^		
Prior to culture	11.32±2.96	-
Following culture	32.65±1.12	-

KPS, Karnofsky performance status; CIK, cytokine-induced killer.

**Table III:** Flow cytometric analysis of changes in lymphocyte subsets and CD4^+^/CD8^+^ratio (means ± SD).

Lymphocyte subset	Observation group (%)	Control group (%)
CD3^+^		
Prior to treatment	70.5±2.45	71.5±5.79
Following treatment	77.5±4.59	70.0±2.97
CD3^+^CD8^+^		
Prior to treatment	35.5±3.57	35.0±2.76
Following treatment	39.5±4.94	34.5±3.87
CD3^+^CD4^+^		
Prior to treatment	34.0±5.46	33.8±6.42
Following treatment	36.0±5.74	32.0±4.90
CD16^+^CD56^+^		
Prior to treatment	11.0±4.98	11.0±3.08
Following treatment	11.5±2.97	9.5±2.31
CD4^+^/CD8^+^ ratio		
Prior to treatment	1.13±0.67	1.21±0.16
Following treatment	1.30±0.56	1.25±0.27

SD, standard deviation.

## References

[1] Linn YC, Hui KM (2010). Cytokine-induced NK-like T cells: from bench to bedside. J Biomed Biotechnol.

[2] Yamazaki H, Shiomi H, Tsubokura T, Kodani N, Nishimura T, Aibe N, Udono H, Nishikata M, Baba Y, Ogita M, Yamashita K, Kotsuma T (2011). Quantitative assessment of inter-observer variability in target volume delineation on stereotactic radiotherapy treatment for pituitary adenoma and meningioma near optic tract. Radiat Oncol.

[3] Wang YS, Wang YY, Jiang P, Ma JJ, Qu Z, Wang XL, Li JT, Jia XF (2012). Short-term outcomes of CyberKnife therapy for advanced high-risk tumors: A report of 160 cases. Exp Ther Med.

[4] Wang CC, Floyd SR, Chang CH, Warnke PC, Chio CC, Kasper EM, Mahadevan A, Wong ET, Chen CC (2012). Cyberknife hypofractionated stereotactic radiosurgery (HSRS) of resection cavity after excision of large cerebral metastasis: efficacy and safety of an 800 cGy × 3 daily fractions regimen. J Neurooncol.

[5] Verneris MR, Baker J, Edinger M, Negrin RS (2002). Studies of ex vivo activated and expanded CD8^+^NK-T cells in humans and mice. J Clin Immunol.

[6] Schmidt-Wolf IG, Negrin RS, Kiem HP, Blume KG, Weissmen IL (1991). Use of a SCID mouse/human lymphoma model to evaluate cytokine-induced killer cells with potent antitumor cell activity. J Exp Med.

[7] Schmidt-Wolf GD, Negrin RS, Schmidt-Wolf IG (1997). Activated T cells and cytokine-induced CD3^+^CD56^+^killer cells. Ann Hematol.

[8] Schmidt-Wolf IG, Lefterova P, Mehta BA, Fernandez LP, Huhn D, Blume KG, Weissman IL, Negrin RS (1993). Phenotypic characterization and identification of effector cells involved in tumor cell recognition of cytokine-induced killer cells. Exp Hematol.

[9] Nishimura R, Baker J, Beilhack A, Zeiser R, Olson JA, Sega EI, Karimi M, Negrin RS (2008). In vivo trafficking and survival of cytokine-induced killer cells resulting in minimal GVHD with retention of antitumor activity. Blood.

[10] Wang X, Wang YY, Jiang P, Ma JJ, Qu Z, Liu HC, Wang SS, Wang YS (2012). Clinical application of CyberKnife for high-risk central nervous system tumors: A clinical trial report of 60 cases. Exp Ther Med.

[11] Rutkowski MJ, Bloch O, Jian BJ, Chen C, Sughrue ME, Tihan T, Barani IJ, Berger MS, McDermott MW, Parsa AT (2011). Management of recurrent intracranial hemangiopericytoma. J Clin Neurosci.

[12] Cengiz M, Özyiğit G, Yazici G, Doğan A, Yildiz F, Zorlu F, Gürkaynak M, Gullu IH, Hosal S, Akyol F (2011). Salvage reirradiaton with stereotactic body radiotherapy for locally recurrent head-and-neck tumors. Int J Radiat Oncol Biol Phys.

[13] Kim HM, Lim J, Yoon YD, Ahn JM, Kang JS, Lee K, Park SK, Jeong YJ, Kim JM, Han G, Yang KH, Kim YJ, Kim Y, Han SB (2007). Anti-tumor activity of ex vivo expanded cytokine-induced killer cells against human hepatocellular carcinoma. Int Immunopharmacol.

[14] Gütgemann S, Frank S, Strehl J, Schmidt-Wolf IG (2007). Cytokine-induced killer cells are type II natural killer T cells. Ger Med Sci.

[15] Weng DS, Zhou J, Zhou QM, Zhao M, Wang QJ, Huang LX, Li YQ, Chen SP, Wu PH, Xia JC (2008). Minimally invasive treatment combined with cytokine-induced killer cells therapy lower the short-term recurrence rates of hepatocellular carcinomas. J Immunother.

[16] Olioso P, Giancola R, Di Riti M, Contento A, Accorsi P, Iacone A (2009). Immunotherapy with cytokine induced killer cells in solid and hematopoietic tumours: a pilot clinical trial. Hematol Oncol.

[17] Maluf PJ, Michelin MA, Etchebehere RM, Adad SJ, Murta EF (2008). T lymphocytes (CD3) may participate in the recurrence of cervical intraepithelial neoplasia grade III. Arch Gynecol Obstet.

[18] Steffens HP, Kurz S, Holtappels R, Reddehase MJ (1998). Preemptive CD8 T-cell immunotherapy of acute cytomegalovirus infection prevents lethal disease, limits the burden of latent viral genomes, and reduces the risk of virus recurrence. J Virol.

[19] Kägi D, Ledermann B, Bürki K, Seiler P, Odermatt B, Olsen KJ, Podack ER, Zinkernagel RM, Hengartner H (1994). Cytotoxicity mediated by T cells and natural killer cells is greatly impaired in perforin-deficient mice. Nature.

